# Cultivating Positive Youth Development, Critical Consciousness, and Authentic Care in Urban Environmental Education

**DOI:** 10.3389/fpsyg.2017.02340

**Published:** 2018-01-15

**Authors:** Jesse Delia, Marianne E. Krasny

**Affiliations:** ^1^Common Ground High School, Urban Farm, and Environmental Education Center, New Haven, CT, United States; ^2^Department of Natural Resources, Cornell University, Ithaca, NY, United States

**Keywords:** environmental education, positive youth development, authentic care, urban environmental education, youth of color, critical consciousness

## Abstract

This paper addresses the issue of how to provide affordances for youth development in the context of environmental stewardship in cities. Urban environmental education encompasses place-based and action-oriented stewardship practices, including community gardening and vegetable production, often with the dual goals of developing youth and community assets. Yet in-depth understanding of how these goals are achieved is lacking. Using narrative inquiry, we explored participant experiences in a multi-year agriculture internship program conducted by the food justice organization East New York Farms! (ENYF) in Brooklyn, NY. Emerging from our conversations with youth were five themes defining their intern experience: ENYF as somewhere to belong, to be pushed, to grapple with complexity, to practice leadership, and to become yourself. We propose a theory of change that emphasizes politicized notions of caring as a foundation for cultivating developmental assets, including competence, contribution, and critical consciousness, among youth who participate in ENYF programs multiple years. This paper extends the literature on socio-environmental affordances to encompass urban environmental education programs, which incorporate physical and social features that act as affordances. Further, this paper describes a feedback loop in which youth afforded opportunities to develop assets through contributing to their community in turn create affordances for additional youth and adults.

## Introduction

Urban environmental education is one means by which children realize the affordances offered by nature and social interactions in cities (Chawla, 2001, 2006). For example, urban environmental education programs serve as settings for developing youth assets (Schusler and Krasny, 2010), elements of sense of place (Kudryavtsev et al., 2012) and social capital (Krasny et al., 2013), and resilience at the individual, community, and social-ecological systems levels (Dubois and Krasny, 2016). In U.S. cities, community organizations and non-profits conduct environmental education programs after-school and during summers, often with youth of color who live in low-income neighborhoods and are hired as paid interns (Smith et al., 2015). Activities include urban agriculture, collaborating with scientists in data collection (e.g., on treatments to mitigate combined sewer overflow), park maintenance, oyster and coastal dune restoration, street tree planting and pruning, and other forms of direct stewardship action. Urban environmental education programs also engage youth in indirect actions such as policy advocacy and teaching younger children (Russ and Krasny, 2017).

In a study of nine rural, suburban, and urban programs that engaged youth in direct and indirect environmental actions, Schusler and Krasny (2010) found that such programs can provide affordances for developing cultural and interpersonal competence, self-esteem, sense of purpose, and other youth developmental assets or qualities that help youth succeed in school and civic life (Benson et al., 2011). However, we lack in-depth knowledge of how features of environmental education programs, such as physical setting and social interactions, afford opportunities for positive youth development in ways that are responsive and relevant to urban young people of color, such as through developing critical consciousness, or a moral stance linked to socio-political awareness and actions in the larger world (Mustakova-Possardt, 1998). In this paper, we explore in-depth how an urban agriculture youth intern program in Brooklyn NY, provides developmental affordances for youth of color in a low-income neighborhood. Specifically, we asked: How do interns' narratives describe the ways in which participation in an urban agriculture, environmental education program fosters development of youth assets and critical consciousness?

Through in-depth interviews with youth, elements of authentic care, or caring relationships that honor students' experiences of class, race, and culture (Valenzuela, 1999), emerged as part of the process of developing youth assets. Thus, we propose that urban environmental education, rooted in a context of authentic care demonstrated by adult leaders and peers, provides affordances for positive youth development and the development of critical consciousness. The results of this research contribute to our understanding of a widespread practice in urban environmental education, i.e., engaging youth in urban agriculture and other direct and indirect environmental action, while expanding on earlier notions of urban affordances for child development, which have focused largely on physical infrastructure (e.g., Kyttä, 2002).

## Theory and background

Research on positive youth development, authentic care, critical pedagogy of place, affordances, and environmental education informed this study and are briefly reviewed below.

### Positive youth development

Starting in the 1990s, interventions and research to support families and children shifted from a focus on problem behaviors of troubled teenagers to factors that are present when youth experience healthy physical, intellectual, emotional, and social development (Eccles and Gootman, 2002; Roth and Brooks-Gunn, 2003; Catalano et al., 2004; Lerner and Lerner, 2011). An outcome of this work is an asset-based approach, referred to as positive youth development, which assumes that all youth have the capacity to become successful adults given appropriate support (Eccles and Gootman, 2002; Lerner et al., 2005).

Positive youth development scholars and practitioners consider both youth assets and the types of settings that enable youth to develop those assets (Eccles and Gootman, 2002). Assets include self-efficacy, prosocial norms, and meaningful relationships with peers and adults, as well as more broadly social, emotional, cognitive, behavioral, and moral competence (Catalano et al., 2004). Another approach to positive youth development focuses on the “Five Cs,” defined as “Competence, Confidence, Connection, Character, and Caring” (Lerner et al., 2005), with competence, confidence, and connections more common outcomes of youth programs than character and caring (Roth and Brooks-Gunn, 2003). Studies of youth programs as contexts for positive youth development have revealed that a sixth C, “Contributions” to community and civil society, is possible when the other five are present (Lerner et al., 2005).

Youth programs conducted by community-based organizations and national non-profits (e.g., YMCA, Boys and Girls Clubs) offer features known to promote positive youth development (Larson, 2000; Larson and Angus, 2011; Lerner and Lerner, 2011; Salusky et al., 2014), including supportive relationships, opportunities to belong, positive social norms, support for efficacy and mattering, opportunities for skill building, structure and safety, and integration across family, school, and community efforts. To support positive youth development, programs should be long-term; foster positive relationships among youth and between youth and adults; include activities that build life skills through setting expectations, posing challenges, and providing recognition; and empower youth by providing opportunities to use life skills as participants in and leaders of community activities (Eccles and Gootman, 2002; Roth and Brooks-Gunn, 2003; Lerner and Lerner, 2011).

Although influential in policy and research, the positive youth development framework has been critiqued for paying scant attention to structural inequities and barriers to development such as poverty, racism, sexism, homophobia, and other forms of injustice (Sukarieh and Tannock, 2011). Social justice youth development, which seeks to cultivate critical consciousness and social action (Ginwright and Cammarota, 2002), addresses these critiques and mirrors calls for environmental education that engages issues of power and justice (Bowers, 2002; Gruenewald, 2003).

### Authentic care

Valenzuela (1999) study of Mexican-American and immigrant youths' schooling points to the importance of authentically caring relationships that honor students' experiences of class, race, and culture. Similarly, Bartolomé (2008) offers the notion of *cariño*, reflecting an “understanding that caring for and loving one's subordinated students is insufficient unless the love and care are informed by authentic respect and a desire to equalize unequal learning conditions in school” (p. 2). For teachers, authentic care goes beyond solely caring for individual students to encompass preparing students to confront inequitable and undemocratic social structures (Ladson-Billings, 1995). Drawing from a study of exemplary black women educators, Beauboeuf-Lafontant (2002) articulates a notion of womanist caring that links self-change to long-term work toward social change, and embraces “the maternal,” political clarity, and an ethic of risk that acknowledges caring does not guarantee one can have a positive influence on youth. Discussions of care and caring in environmental education traditionally focus on teaching students to care for and about nature (Russell and Bell, 1996; Martin, 2007), although McKenzie and Blenkinsop (2006) outline a broader ethic of care in outdoor adventure education programs. Authentic care for youth by adult leaders can be considered a social affordance provided through environmental education programs.

### Critical pedagogy of place

Environmental education taking place in the context of racial and ethnic diversity in cities demands a critical perspective (Ceaser, 2012; Cermak, 2012; McKenzie et al., 2017) that seeks to promote ecological and social justice by addressing issues related to structural oppression and social identity (Cole, 2007). In one such program, Cermak (2012) used “green hip hop” to connect environmental concepts to students' lived experiences as a means of instilling critical ecological literacy.

Drawing on place-based education (Sobel, 2004), ecosystem science, and critical pedagogy (Freire, 1970), Gruenewald (2003) proposes a “critical pedagogy of place” that integrates ecological understanding with an analysis of issues related to power and justice. The goal is to cultivate critical consciousness (*conscientização*, Freire, 1973) and encourage action that addresses poverty, environmental racism, food security, and equitable access to green space. Critical pedagogy of place integrates *decolonization*, or the undoing of the damage caused by oppression through recognizing, developing the tools, and acting to disrupt inequitable systems; and *reinhabitation*, or learning to live well together in a place through restoration, preservation, and transformation of both human and non-human relationships (Gruenewald, 2003; Gruenewald and Smith, 2008). Smith (2007) demonstrated how community-based projects were successful in connecting students to local people and places—supporting a process of reinhabitation—but took only small steps in instigating a process of decolonization, which he attributes to educators' fear of administrator or community backlash when addressing controversial topics.

### Affordances and environmental education

Previous work on affordances has focused on how children in environmental education programs can use and shape features of the physical environment, such as making a sculpture from branches or building a dam with small rocks in a stream (Chawla, 2006; Said, 2012). Being able to use and shape these physical affordances contributes to learning and action. According to Chawla (2006), when children are able to see changes in their environment as a result of their actions, they learn not only about physical properties of the world but also about their own capabilities, and thus develop competence.

In addition to physical settings, behavior settings that enable interactions among youth and adult leaders provide opportunities for learning (Chawla, 2006). Researchers have emphasized affordances for developing social skills, which may be particularly important for children and adolescents as they go through stages of development (Kyttä, 2004), and have described how children might shape affordances through participation in environmental planning (Kyttä et al., 2004; Rudd et al., 2017). Clark and Uzzell (2002) proposed the notion of integrated socio-environmental affordances, such as a young person's home, school, playground, neighborhood, or city center, which integrate physical (e.g., plants, buildings) with social features, such as people with knowledge, observable behaviors, attitudes, and cultural values. We suggest that urban environmental education programs also offer socio-environmental affordances stemming from the rich settings (e.g., community gardens, farmers' markets) and social interactions that occur within these programs. In this paper, we are particularly interested in affordances that lead to the development of youth assets, such as caring, contribution, and competence, and that incorporate critical thinking or awareness.

### Environmental education programs as providers of affordances for positive youth development

Environmental education encompasses a wide range of programs that provide access to nature and adult mentors (Sauvé, 2005; Fraser et al., 2014), including those in which youth are active agents changing their environment and community. Thus, similar to youth programs more broadly, environmental education programs may provide adolescents with opportunities to develop responsibility and agency (Larson and Angus, 2011; Salusky et al., 2014) and may serve as a gateway to broader participation in civil society (Lerner et al., 2003, 2005; Lewis-Charp et al., 2003). In particular, place-based (Sobel, 2004), critical pedagogy of place (Gruenewald, 2003), participatory (Reid et al., 2008; Læssøe and Krasny, 2013), and environmental action (Jensen and Schnack, 1997; Schusler and Krasny, 2014) approaches to environmental education seem well-suited to providing affordances for positive youth development because they engage young people in reflection and in community environmental action.

Insights into positive youth development and environmental education have emerged from studies of environmental action programs in community settings (Schusler et al., 2009; Schusler and Krasny, 2010). Such programs engage youth in volunteerism, service learning, and related forms of civic participation, which provides opportunities for developing youth assets (Lerner et al., 2003; Lewis-Charp et al., 2003; Chung and Probert, 2011; Lerner and Lerner, 2011). In one study, educators leading environmental action programs spoke about “preparing youth for future roles as voters who think critically about issues and as citizens committed to serving their community whether in environmental or other arenas” and as “agents of social change within their communities” (Schusler et al., 2009, p. 117). Other studies have linked youth environmental civic engagement to school success, communication skills, feelings of self-worth, sense of social commitment and responsibility, and development of social skills and positive relationships (Riemer et al., 2014; Stephens, 2015). Leaders of environmental action programs describe multiple program elements that contribute to positive youth development including creating safe spaces, providing structure, building relationships, bridging differences, setting expectations, providing opportunities for meaningful contribution, supporting youth, connecting youth with their community, and expanding horizons through novel experiences and reflection (Schusler and Krasny, 2010). Similarly, studies of environmental clubs have documented multiple positive youth development outcomes in Africa (Johnson-Pynn and Johnson, 2010), China (Johnson et al., 2007), Guyana (Comber, 2016), and Canada (de Vreede et al., 2014).

In addition to being outcomes of environmental action programs, youth assets such as locus of control and social connectivity may predict future environmental behaviors (Hungerford and Volk, 1990) and collective environmental actions (Chawla and Cushing, 2007), suggesting feedbacks between engaging in action and developing assets. The notion of feedbacks between environmental action and positive youth development is consistent with Silbereisen and Eyferth's (1986) “development as action in context,” which proposes that development is an outcome of intentional, goal-oriented action that produces changes not only in the individual but also in the program context or community. For example, youth in environmental action and club programs shape their environment to meet larger social goals—such as planting a community garden to enhance community cohesion and food access—which in turn provides a setting for youth to develop competence and self-efficacy. In short, adolescents in an environmental education program may *shape* an environment or an affordance so that it supports the pursuit of their goals and in so doing develop assets (Clark and Uzzell, 2002).

## Methodology and methods

The results reported here are drawn from a master's degree study integrating positive youth development and critical pedagogy of place in urban environmental education (Delia, 2013).

### Program context

We examined positive youth development within the context of the non-profit organization East New York Farms! (ENYF), whose mission is “to organize youth and adults to address food justice in our community by promoting local sustainable agriculture and community-led economic development^1^.” Because ENYF is part of a community organization started in the 1960s as a grassroots, community response to racist policies and poverty (Thabit, 2003) and a need for educational and social programming (UCC, 2017) in East New York, we felt it would be an ideal site to examine positive youth development within the context of an organization that explicitly integrates resistance and critical perspectives into youth and community development.

We worked with ENYF's urban agriculture youth internship program, which employs up to 35 youth during the growing season from March until November. The interns learn hands-on agricultural skills at the ENYF farm and in nearby community gardens, run ENYF farmers' market stands, and learn about environment, health, community development, leadership, and social justice through their hands-on experiences and workshops. First-year interns participate in all aspects of growing and selling the food at markets as well as in all in-house workshops and off-site conferences. Returning interns (second-, third-, and fourth-year) take on greater responsibility including leading crews of first-year interns at the ENYF site and nearby community gardens, learning specialized knowledge (e.g., about producing and selling food), and leading workshops for first-year interns and at conferences (Delia, 2013). In this study, we focused on nine returning interns some of whom were also crew leaders for younger interns, and how they depicted the ways in which participation in an urban, farm-based environmental education program contributed to their development and critical consciousness.

### Methodology

The first author used narrative inquiry (Clandinin and Connelly, 2000) over two summers to establish a trusting relationship with ENYF staff and youth, to understand how youth and staff perceived the youths' experience at ENYF, and to offer insights that can be reshaped and applied in other settings. Narrative inquiry is suited to the study because it allows for deep understanding of participants' experiences in the context of their lives within and beyond ENYF, and addresses ethical considerations and views of the authors about what counts as knowledge and the purposes of research (e.g., that research “subjects” are authoritative experts on their own lives and lived experiences, and that this knowledge goes beyond the “anecdotal”). Reflecting “the stories of life contained in the inquiry” (Clandinin and Connelly, 2000, p. 41), the interns' personal narratives allowed us to explore in-depth observations and practical theories regarding positive youth development in the context of an urban agriculture intern program.

### Ethics

We followed an appreciative inquiry process, defined as “a research method focusing on positive organizational attributes that may fuel change” (Grant and Humphries, 2006, p. 402), to address ethical concerns about conducting research on a small, community-based organization and its participants. This entailed focusing our interview questions on youth assets and programmatic strengths to elicit intern stories that demonstrated what worked well at ENYF, while applying a critical lens through reflection and deliberation during (Reed, 2007) and after (Grant and Humphries, 2006) the inquiry process. Further, recognizing her positionality as a white outsider, ten years older than interviewees, the first author and researcher spent significant time participating in the program to be able to develop trusting relationships with participants. She sought to bring intern voices to the forefront and allowed interns to largely determine the tempo and direction of the interview. Finally, the first author is an experienced youth worker who brought training and expertise to engaging young people in conversations around potentially challenging topics.

This study was approved by the Cornell University Institutional Review Board and ENYF. Informed written consent was obtained from all interviewees and where appropriate, their legal guardian. In the consent form and in practice, participants could decline to participate in the study, refuse to answer a question, or request to turn off the audio recorder at any time.

### Participants

After working alongside interns during the first year to establish trust and rapport and develop preliminary insights, the first author pursued open-ended interviews with nine returning interns (two males and seven females). The interviewees were purposely chosen as being the only returning interns in the program and thus being able to reflect on their long-term internship experience. All interviewees were secondary-school-aged (15–18 years, mean age = 16.3, *SD* = 1.1) youth of color living in Brooklyn, NY. Their ethnicities and race varied, including African-American and youth of Dominican, Puerto Rican, Nigerian, Guyanese, and Jamaican heritage. Family income for interns was not available although ENYF considers financial need in the intern application process. All names used in the study are pseudonyms chosen by the participants to protect their anonymity.

### Data collection

In her first field season, the first author conducted informal discussions and focus groups with interns and ENYF staff to develop the interview questions. During the second season, she conducted and recorded two-part interviews (each about an hour in length) with returning interns to elicit thick description (Geertz, 1973) of participants' ENYF experiences in the context of their personal histories. The first interview, designed to understand youths' stories in context, focused on life-story and personal background, prioritizing questions related to school, family, friends, and role models, activities outside the program, and future personal and professional aspirations. The second interview focused on participants' ENYF experiences including how they found out about the program; how they became involved; stories about their interactions with other youth, staff, and community members; and reflections on what they had learned and its usefulness outside the program. All interviews were conducted one-on-one with the exception of one second interview conducted with two interns. The first author also recorded reflections and observations of the intern program in field notes.

### Data interpretation

The first author and two research assistants transcribed all the interviews as close to verbatim from the recordings as possible, including various speech utterances (e.g., um). The first author then read all transcripts, which informed her initial understanding of the relationship between the two a priori theoretical frameworks (positive youth development and critical pedagogy of place) and the emergent focus of care and caring. During subsequent coding, the first author focused on themes that emerged from analysis of individual narratives, which she then “checked” and explored further by looking across narratives.

Although the a priori frameworks influenced the initial reading, subsequent interpretation was based on open coding of the narratives. By utilizing both deductive and inductive strategies, both authors were able to identify codes that were informed by questions related to positive youth development and critical pedagogy of place but also be open to emergent codes that connected statements in context of a coherent understanding of the whole program (Glaser and Strauss, 1999; Maxwell, 2005). After the first open coding generated an extensive list of codes, the first author revisited the data to identify major themes and subthemes related to these codes using Atlas.ti. Through an iterative process of reading, coding, and reflecting on the transcripts, a relationship between the major themes emerged, which the authors sought to represent as a theory of change to explain how ENYF fosters positive youth development.

The first author returned to ENYF to conduct a focus group with a subset of interviewees to solicit feedback on early data interpretation. She shared transcript excerpts with participants to member check their stories and seek permission before sharing quotes. Through discussions with ENYF staff, she also developed a deeper understanding of the emergent theme of authentic care.

Throughout the data interpretation, the first author recorded memos noting general themes, patterns, novel or confusing information, and contradictions within and across narratives (Saldaña, 2013). She also used a process described as “rigorous improvisation” in the context of social justice youth development research (Ginwright and Cammarota, 2007), which involved observing young people at ENYF, immersing herself in dialogue with academic colleagues about what was happening in the program, returning to ENYF to delve deeper into understanding youths' experiences, and revising her interpretation based on overlapping and divergent information within interviews, literature, conversation, observations, and ethnographic notes.

## Results

… you not dragging me down now ‘cause I got somewhere to *go*, I got somewhere to *be*, so when you gonna stay in this house, while I go work at the garden, at the farm, you know, making my money while you just sit here. (Nova, fourth-year intern).

Starting with Nova's statement in which she passionately explains how ENYF gives her “somewhere to go…somewhere to be,” we explore how ENYF is not just a job or educational program for the interns, but *somewhere to belong, to be pushed, to grapple with complexity, to practice leadership*, and *to become yourself*. Within each of these themes that emerged from our analysis, we identified subthemes, which we mapped to constructs related to developmental assets and settings from the positive youth development literature and to critical consciousness from critical pedagogy of place scholarship (Delia, 2013). Below we cover all five themes but focus on *belonging* and *being pushed* as relates to authentic care, and *grappling with complexity* as relates to critical consciousness. We have edited the intern quotes illustrating these themes slightly to remove words such as “like” and repeated phrases, while trying to maintain youth voice accurately. A full narrative account of the interns' stories can be found in Delia (2013).

### Somewhere to belong

…so we see that person—all right, they havin' a bad day. And we go cheer ‘em up or go talk to them… It's just like a family. East New York Family Farm—like a family. … I'm wanted here ‘cause I don't wanna be no place that I'm not wanted. (Cedrick, second-year intern, crew leader).

Youth shared stories of their initial days as first-year interns and how they remembered *feeling welcomed* and appreciated by staff and returning interns. Over time, interns began to *feeling safe* and able to open up to adults and their ENYF peers, and *felt cared for and connected*.

#### Feeling welcomed

Tamara recounts her early days at ENYF and the ways in which other youth persisted in their attempts to welcome and get to know her when she was reluctant to open up.

My first year, the first time I started working here I was quiet, like I didn't want to talk to nobody. I didn't really know anybody and I was working in the garden and then I had to work with Kimberlé, Jayden, and Aisha… Jayden kept trying to get conversations out of me. And I was answering him with one [word] answers. … and then Kimberlé tried talking to me and then she was like, you know, forget it. And then, everybody, but they still trying to talk to me and then finally I loosened up and I started talking. (Tamara, third-year intern, crew leader).

Kiah explains how staff members made her and the other interns feel respected and appreciated when they first started the youth program.

… when I first came to the program, [staff member] made me feel welcome… even when I wasn't really open—I wasn't really open to like a lot of youth here, she always made me feel comfortable, you know, like I was appreciated or something. (Kiah, second-year intern).

#### Feeling safe

Kimberlé recounts a story in which she shared personal information with a staff member, explaining how the staff person would never “throw her under the bus” or break her confidence. She also makes a connection to a feeling of safety and familiarity by describing ENYF as a kind of second home.

…one person I always go to…just to talk and get a lot of stuff outta my mind is [staff member]. Like, she's a good listener, and she just, I really talk to her about anything and everything, … you can come in, and if you have something on your mind…you can talk to them about anything. …they wouldn't you know, throw you under the bus, … look at you a different way because, you know, ohh, you talked to them about something… they wouldn't spread your business also, so yeah, that's another good thing about this job. It's very comforting, you know, you feel like it's a second home. (Kimberlé, fourth-year intern).

#### Feeling cared for and connected

Returning interns described the ways in which their bonds with other interns and staff knit them into a family complete with inside jokes and stories, nicknames, favorite “siblings,” and an experience of intimately knowing and being known. Tamara described the ways in which “people in this job notice stuff about you,” giving the example how she earned her nickname “Miss Buttercrunch T” for her love of buttercrunch cookies. She also articulates how other interns and staff observe and adopt each other's unique forms of expression, similar to how family members share mannerisms, speech patterns, and gestures. By pointing out, riffing on, and even adopting one another's idiosyncrasies, the interns and staff are drawn closer together.

… oh gosh–who could not talk about Kimberlé's laugh? …you can't meet somebody with a laugh like Kimberlé's. … and now it's contagious…Saturday I was working, I was painting and Priscilla was doing market, she was doing the stand and she laugh and they said, “oh gosh, you can hear Kimberlé all the way in the garden.” And I was like, that wasn't Kimberlé! That was Priscilla. (Tamara, third-year intern, crew leader).

Cedrick explains how paying attention and getting to know the other interns actually allows them to “bring each other up,” make each other feel better, and look out for each other at work, pointing to the ways in which supporting peers is a learned skill. Here he talks about Sadie, his fellow returning intern and co-crew leader.

Sadie [is the] person [that brings me up] ‘cause there's supervisors but me and Sadie knew each other from last year. …me and Sadie got a connection that nobody expect ‘cause the returning interns all got connections that people might not understand. You might look at each other, start laughing. They be like, “what is wrong with y'all two?” You be like, nothin'. It's just something that we remembered. So…we all got the connection that always bring each other up. Like everybody knows when I'm mad ‘cause I'm quiet. Everybody knows when Tamara's mad ‘cause she gets that face. Everybody knows when Schuyler's mad ‘cause Schuyler goes in the hallway. So it's like we all know each other already… (Cedrick, second-year intern, crew leader).

Returning interns' stories made clear the importance of close, caring relationships with staff and other youth. In this sense the staff's work with the youth and in turn the returning interns' work with first-year interns became an affordance that enabled the youth to develop caring relationships.

### Somewhere to be pushed

…[staff] really push you a lot … it's just so you can have fun and break outta your shell, you know? So that's really good about this job. (Kimberlé, fourth-year intern).

Returning interns share stories about how staff demonstrate *high expectations* by challenging them to perform new roles, revealing how they negotiate the tension between the discomfort of being asked to do too much and the benefit from performing outside their comfort zones. They also explain how the *clear structure* of the program pushes them to honor the guidelines and agreements they make at the beginning, pointing to both the frustration and appreciation created by rules and the consequences of not following them. Interns struggle early on but later thrive within this program structure, which initially holds them accountable for their behavior and over time facilitates them taking ownership for their actions.

#### High expectations

Kimberlé tells a story about how the staff pretended not to know the routine in order to press interns to take leadership at the farmers' market: “during the markets, [the staff] would catch amnesia and they wouldn't remember, we would have to clean up the market without going to them.” She goes on to explain how the staff's high standards pushed her and others to grow or to “crack you out your shell.”

… other jobs, they don't really do what, you know, this job does. Like this job… you get a learning experience, you go to workshops…they teach you a whole bunch of stuff about leadership, dedication, how to save your money, everything…they don't only send us out to the garden, we have a farmers' market, we have tours, we have, just have a whole bunch of stuff. (Kimberlé, fourth-year intern).

Sometimes the high expectations of staff can be overwhelming, as Tamara articulates.

Maybe sometimes it can be a little bit too hard on us …I think sometimes they expect us to … know everything. … Sometimes we feel like we're stuck in between a rock and a hard place and we don't know what to do because, you go to them, and ask for help… and they're like, well, you should know that and it's like you go and you do it. (Tamara, third-year intern, crew leader).

Tamara explains how staff pushed her and others to stretch out of their comfort zone and into new roles leading younger interns:

(w)hen I had to first lead the harvest by myself… some of the kids would ask me, “How do you do this?” and I was like, I don't know. … when I first did my first harvest I think I had Sadie with me and I was asking Sadie and she was like, “I don't know.” We both … was like, okay, go over there and ask [staff member]. …sometimes she would give them an answer and sometimes she would send us back to them and we were like, dang. She sent them back.

Kiah appreciated the challenging expectations: “I appreciate every task that I get because I see them helping me…for the real world.”

#### Clear structure

ENYF staff make the program structure transparent to interns from the start of the season. Interns sign contracts agreeing to specific guidelines with consequences for “violations” including reduced wages. They also agree to participate in a system of straight talk with staff and peers that includes receiving positive affirmations and constructive criticism on their work performance. The clarity and enforcement of the expectations and ongoing feedback provide opportunities for youth to improve their work and leadership skills. Youth interns struggle initially but many thrive within a system that holds them accountable for their behavior. Several even offered feedback to the first author that she should more strongly enforce the guidelines to help meetings go more smoothly.

Kimberlé explains the intent and consequences of violations and how the system supports the youth. Even during a challenging time where she sometimes just felt “down,” she kept a mature perspective about the purpose of violations as helping interns identify areas for improvement.

(v)iolations are not really, to like, throw you under the bus or throw you down. But it's really to … be like, “Hey, you know. You did this, can you improve on it.” … unless it's to the certain extent where you're cursing and fighting, you know then… you need to change your act, that's when the strictness comes. …there'll be some days last year I used to get unmotivated, … where I just was like “You know, I'm just down,” you know? And…it's just really to better yourself, and not really to, you know, throw you down. (Kimberlé, fourth-year intern).

Cedrick describes how the system for violations becomes more rigorous for returning interns who can no longer “earn back” or reverse the impact of a violation in the way allowed for first-year interns.

I miss those days, like first-year, you can't do no wrong. It was like, you got earn-backs so you get a violation, take five dollars out of your paycheck … Next paycheck, you got five dollars back on your paycheck. It's like, whoa, ok, earn-backs. This year, no earn-backs. You like, “Oh. What happened?” So it's different. I still love it. (Cedrick, second-year intern, crew leader).

The program structure is daunting at first but like most returning interns, Cedrick rises to the occasion and plans his daily routines such that he is motivated, on time, and prepared each day for work.

### Somewhere to grapple with complexity

I like seeing direct change and immediate change but that's not always the way things happen, sometimes it's a long change. Yeah, like we was trying to talk to people and stuff like that but some people didn't wanna learn about something that was new… they were so much brainwashed throughout their life, that they just don't accept no more information. They're like, “Aw, someone already told me information. My mother told me that this was the correct way and how are you a stranger telling me that that's the wrong way?” So, yeah telling people, you don't have to always stay with something. You could always learn new things and if you think it's correct, you could go with it. (Sadie, second-year intern, crew leader).

Returning interns spend much of the growing season grappling with *complex tasks* including leading crews of first-year interns, giving and receiving straight talk, managing farm and farmers' market responsibilities, serving food at a soup kitchen, and speaking publicly at workshops, conferences, and occasionally public hearings. They also grapple with *complex concepts* related to the environment, ecological systems, and questions of food justice and the food system at workshops and conferences. While returning interns may not report every detail accurately, they do attempt to articulate what is important about the concepts they are learning and the work they do. Most notably, returning interns express pride at learning through challenges and demonstrate an emerging critical consciousness through their questioning of what they are learning.

#### Complex tasks

Returning interns take on new roles (e.g., crew leader, Urban Agriculture Intern) that involve managing and completing complex tasks. As a crew leader, Tamara explained the myriad day-to-day responsibilities of picking a game and opening ice breaker question for her crew, keeping track of time, getting outside, and remembering to put away all the tools. Crew leaders also manage weekly harvests with the first-year crews.

Kiah is responsible for managing the share table, which involves collecting and tracking multiple gardeners' produce for sale. At the farmers' market, all sales and amounts must be recorded based on whose produce is sold, a complicated job that can be compounded by latecomers dropping off their produce on market day. Kiah masters this task and is able to take on new projects, which she sees as helping her prepare for the future.

…as far as my job go…it's mad stressful ‘cause you come in, you go to the garden to whoever garden you goin' to, you get they stuff, you record it on the list, then after you record it on the list, you come back here, you gotta wash it and weigh it, then after you wash it and weigh it, put it in the fridge. Make sure you put labels for their names, so that way they don't get confused. Then it's like, all right, boom! (claps) You done. Whoo! Now Saturday come, now people wanna come with they stuff, mad late. I ain't gonna put no names out, but a few … people be coming mad late and stuff and then it gets really confusing when they come at you… (Kiah, second-year intern).

In addition to their daily responsibilities, returning interns give “straight talk” to other interns and staff at the end of the season. Although returning interns are accustomed to receiving feedback through straight talk, giving comments presents the challenge of communicating constructive criticism to peers and supervisors, which feels complicated because youth worry about making others feel badly. As Kimberlé commented, “it's very difficult to give comments when you're really close to someone.”

#### Complex concepts

In addition to job responsibilities, participating in and teaching workshops challenges interns to grapple with complex environmental, food systems, and social and food justice concepts. Returning interns work to articulate the multifaceted nature of these issues; while at times their explanations are incomplete, they demonstrate critical questioning as well as an emergent critical social-ecological consciousness.

Sadie describes a workshop in which she has to practice talking to others about the importance of behaviors such as composting. In addition to beginning to understand ecological processes, she is thinking about why teaching others about environmental behaviors is important.

Like how to talk to someone like, when you cross the street, get your point across and they would know what you was talkin' about. So that helped me, you could start off the conversation like, “Oh, do you know that mostly all of our leaves go to the landfill in New York, New Jersey and all of that could be composted and put into nutritious dirt, for the soil and it's going right back into the earth. It's like a cycle process.” And they're like, “Oh, that's true, I…” and then they're like, “I just threw a bag of leaves in the garbage.” I was like, “See, that could have been right here, you could even put it like in a jar or something and bring it to us,” and they be like, “Okay, so next time I'll rake my leaves and bring it.” I was like, “Yes, that's a good idea.… That grass could go in compost too. You don't have to mow it on the streets and then it goes into ocean and then it just sits, stays there just like seaweed.” (Sadie, second-year intern, crew leader).

Sadie's discussion of vegetable production demonstrates how she is grappling with understanding and articulating food system issues and opportunities.

… a cucumber from California and a cucumber from East New York … the one from California was waxed and the one here wasn't, it was just shiny naturally and theirs was extremely big which you know they were inputted with steroids and stuff like that. Ours was a good size but it wasn't like there was some fertilizer that wasn't organic. And we learned the farmer gets one cent per tomato and we pay 50 cents a pound for a tomato and how does the farmer get one cent? … so it taught me you should really think about who to buy from, a lot ‘cause the gardeners, you're paying exactly what they're selling. They're givin' it to you and you're givin' the money back to them so it's like healthily grown instead of all the way from half around the world and all the pollution that has to come over here, and all the workers that it's payin' minimum wage or even lower or the immigrants that was forced, “if you don't do this, I'm gonna take you to the immigration center.” (Sadie, second-year intern, crew leader).

Similarly, Schuyler works to articulate her understanding of the way food system issues including eating healthy will take “years and years and years to fix because it took years and years to build.” She reflects on the ways in which her job at ENYF has helped her learn about “the things that we deal with every day that you can actually be blindsided by” and the impact of an unjust food system on people in her community. When asked to give an example of what she meant by being “blindsided,” Schuyler told a story about a workshop in which she learned how much sugar is in an Arizona iced tea.

The workshop with … the Arizona. I used to always buy Arizona's, and I'm like, I'm looking at the Arizona when they show me how much sugar is in Arizona. I was like, that is ridiculous. There is no reason why there should be that, the whole bottle should contain so much sugar. And the fact that they're actually tricking people in the bottle, saying, oh it's such and such grams for 8 fluid ounce bottle but the bottle itself is 20-something ounces, 20-something fluid ounces. And I was like, that is ridiculous. (Schuyler, third-year intern).

She then shares her struggles with how to personally address health and food issues.

Ever since I was younger, I used to always be a person to love food but I'm trying too hard to start eating more healthier, I guess you could say. But then again, if I get hungry, and I don't feel like cooking or something, I'll be the first one to run to the corner store and get a bag of chips or something like that. And it's something I'm accustomed to, and, to be accustomed to something, it's really hard to get away from it. So, …the knowledge that I have now is helping me a lot to be able to understand what's going around… what is actually happening in the community. But, like, habits and being accustomed to different things is making it harder for me to actually adapt to the knowledge that I just learned about the community. So, even though–all the knowledge I have about this stuff, I don't even know if I'll be able to use it. (Schuyler, third-year intern).

Schuyler's descriptions are rich, complicated, and personal. She sees that change will take a long time and that adapting to new knowledge can be difficult. Whereas she might not offer an answer to solving food system problems, she is asking critical, systems-level questions as she works to make changes in her personal life.

### Somewhere to practice leadership

I feel like this program really shook the shy outta me. (Kimberlé, fourth-year intern).

Returning interns reported the ways in which they learned about *responsibility and accountability* by growing into new positions and leadership roles within the program, and how they *became the experts* at ENYF and beyond by leading workshops and volunteers and by teaching others outside of the program. Finally, they shared their reflections on leadership and their *developing praxis*, while stressing the importance of *youth as leaders* in the food justice movement.

#### Responsibility and accountability

Returning interns talk about having responsibility for teaching new interns because older interns previously taught them, and about learning how to be responsible for their own actions and those of their crew. For example, Schuyler talks about learning to take responsibility for miscommunication between staff and interns.

…if I work on a project, before, I'd probably be “Oh, she made that mistake”… But now I'd probably be like, Oh, it was a team effort that we both take responsibility for the mistakes that we made. And I will be able to be more accountable for others because, I guess I can say I struggled with that before. Like if I messed, if I was working with a team and I messed up, I'd be the first one to point, “I did not do that! That was her!” So I guess now I'll able to account, to have accountability for others. (Schuyler, third-year intern).

#### Becoming experts and teaching others

Returning interns teach and become a role model for younger interns, and share their new knowledge with ENYF adult volunteers, thus becoming a “parent of the garden.” They also share their knowledge with parents and peers at workshops outside of the program. Gloria explains how she is struggling to overcome nervousness about offering her ideas.

I think one of my weaknesses is keeping things to myself, like keeping things bottled up inside. …maybe when I have an idea, I'm not like the first one to say “Oh, I have an idea!, like, here's a faster way to do this”–and I think trying to be a good leader, I haven't yet mastered that yet. This program is definitely helping me but, like it's so hard! (laughs) ‘cause, you know sometimes you're skeptical about what people will say like, “Would this really work out?” or “What is she thinking?” … Maybe that's one of my weaknesses but, I should just not care about what people think, and just say it, or, voice my opinions sometimes. (Gloria, third-year intern).

#### Developing praxis

Through their experiences, returning interns recognize leadership as a process rather than a discrete accomplishment. Cedrick reflects on how he works with younger interns.

I don't want [a new intern] to feel like, “Oh, that person's bothering me. I'm not coming to work.” Messing up an opportunity, because somebody's bothering ‘em. That's what I always tell the first-years. “If you feel somebody's playing too much, come to me. If I'm not in your group, go to your crew leader.” (Cedrick, second-year intern, crew leader).

Additional elements of Cedrick's developing practice include “crackin' jokes” with his crew to break down “walls” or communication barriers within the ENYF “family.” Other returning interns talk about the importance of “straight talk” or feedback in helping them to hone their leadership skills.

#### Youth as leaders

Returning interns reflect on how by doing community food work and by being leaders in their community, they are defying stereotypes of what teenagers are “supposed” to be. They also express awe and inspiration at attending conferences in which youth take active leadership roles. Amazed by youth coming from all over to a conference in Philadelphia, Cedrick sees that he is part of a food justice movement larger than himself or ENYF, and how he might benefit from the connections he's making.

It was like…now I know I'm not the only person doing it. I know different people, around New York City, in the United States is doing it, so I'm like, okay. People from Cali, Chicago, Louisiana, North Carolina, Kentucky, Texas, Boston, it was a lot of different people… you made a lot of friends, you might see in the future, that might help you, in different ways. Be like, Oh, remember me? Can you do me a favor? So it's a lot of people, it's good, knowing a lot of people.

… at the conference. Youth bill of rights…food justice, we want all food to be organic, we want at least five pounds in each store of fresh fruits and vegetables from the community, so it was all about food justice. It was just saying what we demand, as youth and everything, to make sure that our generation, and the next generation doesn't suffer from the generation that's in front of us. So it's like we just want a healthy and better lifestyle than other people. (Cedrick, second-year intern, crew leader).

### Somewhere to become yourself

Well, I feel like you learn to gain… confidence in yourself. Not to always be shy because that's how I was when I first got in here. Like I was just so shy and quiet… But yeah, this program really helps you to shaping yourself up. Learn a lot of stuff, gain a lot of knowledge about the garden and the fruits and vegetables and, during the protest [in support of community gardens], even though I was nervous, I just shook it off, and you know, I'm doing it for a right cause. So yeah, it was really exciting. And you just really learn how to just, you know, be yourself. (Kimberlé, fourth-year intern).

Returning interns tell stories that reflect the development of a *new sense of self*, or “re-storying” who they are. Although this can be expected as youth become young adults, returning interns also point to a *triumph over the past* and their possible future. Out of this triumph comes the choice to pursue a particular decision or even major life course; most striking in these decisions is that returning interns now have the knowledge and ability to define and pursue *success on their own terms*. These three subthemes are integrated in the quotes below, starting with Kimberlé explaining overcoming odds to succeed in the intern program and in school.

…another success is being here for my third year, looking back to like my first year, it's really successful … getting accepted to a college … my one main big thing was getting my [high school] diploma, …, I was so happy when I went back to school on Tuesday, and she handed my diploma and I put it in my case…I'm so amazed that I really made it. ‘Cause you know, my sister, she didn't make it, my other brother didn't make it, … so I'm the first one who has a diploma in the family. (Kimberlé, fourth-year intern).

Tamara explains how working with diverse youth and the ENYF guidelines around confidentiality help her to learn to trust and confide in others, which is important given her previous experiences.

I never used to trust people and I always like to be by myself, wanted to do everything on my own, I didn't want no help, nothing. And then coming here and having to work with another person and they havin' to help me like, shovel something or doing somethin' that had to be worked in a pair and then having to trust that the person's gonna do it and I think—giving trust is easier for me … now and… it helped me out because if I needed somebody to talk to I know I can always run to one of my other co-workers or one of the supervisors and talk to them and let them know something and know that I won't have to hear it back another minute. (Tamara, third-year intern, crew leader).

Finally, Sadie reflects on how adults in the community are taking note of the changes interns have made in “re-storying” themselves.

… we're all changing like the way people see teenagers. Instead of being rude, we're trying to help the community and being respectful. It's like adults and all. So I was like, this is good so that people don't always stereotype us… (Sadie, second-year intern, crew leader).

## Discussion

Our results suggest that in addition to nature and neighborhoods, an environmental education program that engages youth in environmental action can provide socio-environmental affordances for positive youth development. Whereas ENYF's urban farm, community gardens, and farmers' market constitute physical settings that provide affordances (e.g., through youth growing produce, managing produce sales), the interns' stories place greater emphasis on the social affordances contributed by adult leaders and other interns. In particular, the interns spoke about how adults create a sense of belonging, yet constantly challenge them to perform complex tasks at the farmers' market and to grapple with complex concepts during workshops. This fosters interns' developmental assets including caring for and connection to other youth, adult leaders, and the broader community; and competence in performing tasks and in beginning to develop critical consciousness. Whereas connection and competence are commonly seen as outcomes of youth programs, previous work has shown that caring (Roth and Brooks-Gunn, 2003) and critical consciousness (Smith, 2007) are more difficult to cultivate.

Developing these assets occurs through youth contributing to the intern program (training younger interns) and to the broader community (community gardening, farmers' market, workshops); in this way, intern interaction with the ENYF program affordances creates new affordances for interns and the community. Further, in addressing and developing a critical awareness of food system justice, youth become agents of change. Through their contributions, ENYF youth develop critical consciousness and the program reflects a social justice youth development approach (Ginwright and Cammarota, 2002, 2007; Ginwright and James, 2002).

Below we first articulate a theory of change that emerged through our work at ENYF, and then discuss connection and caring as relates to authentic care, competence as relates to critical consciousness, and contribution as relates to creating new socio-environmental affordances for youth and community development in cities. Because other urban youth intern programs may be seen more as summer jobs and provide fewer opportunities for youth development (DuBois et al., 2017), the program model and discussion are specific to ENYF yet provide insights into what is possible through thoughtfully designed programs.

### Program model

**Figure 1:**
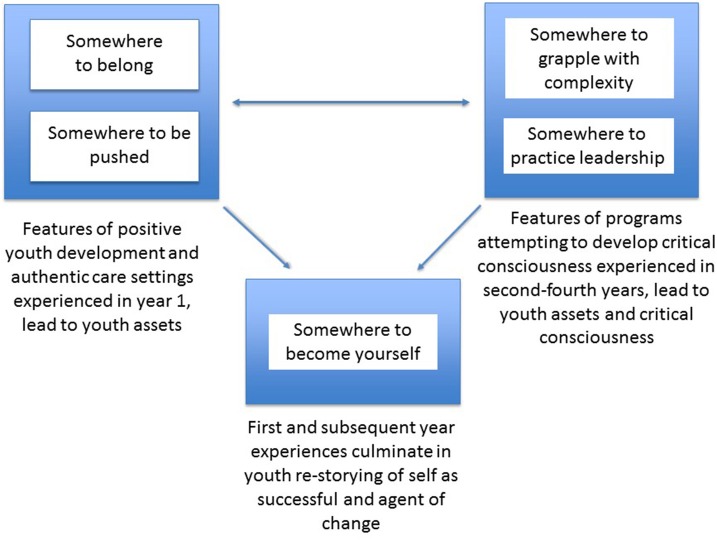
Program elements can be considered affordances for positive youth development for interns, provided initially by staff and later by returning interns themselves.

ENYF interns' testimony illustrates how a participatory, praxis, place-based, and critical form of environmental education, known as environmental action (Schusler et al., 2009), provides socio-environmental affordances for youth development in an urban community. The process (Figure 1) begins when educators create *somewhere to belong* as characterized by interns' stories of feeling welcomed, cared for, and safe. But it is clear from the expectations educators set at the onset that ENYF is also *somewhere to be pushed* and youth soon come to realize that the program entails taking on difficult challenges. Creating such a sense of belonging while presenting challenges are features of positive youth development settings (Eccles and Gootman, 2002) and of authentic care (Valenzuela, 1999).

Returning interns also find that the program becomes *somewhere to grapple with complexity*, building on their first-year experience and being asked to perform ever more challenging tasks. They are also challenged to think through contested social and environmental justice issues, and thus begin to form a critical ecological literacy (Cermak, 2012). In addition, returning interns delegate responsibility during harvest time and model and reinforce community norms while facilitating workshops; in this way the program becomes *somewhere to practice leadership*. The challenges staff present to the interns (e.g., “amnesia” about tasks they feel the interns can tackle on their own) are consistent with findings demonstrating how leaders of youth environmental action programs continually struggle with how to balance offering youth guidance while letting youth take the lead (Schusler et al., 2017). Further, similar to youth in other environmental and social justice programs (Fusco, 2001; Calabrese Barton, 2003), returning interns demonstrate leadership situated in and for community at ENYF and in the broader community, as they teach peers and community members what they learned. Finally, as youth come to see ENYF as *somewhere to become yourself*, they draw on their skills, experiences, critical reflections, and relationships to create a story of their future, defining success on their own terms. In short, the affordances provided by ENYF come in the form of interns practicing community environmental stewardship, while building relationships with and learning and teaching alongside their peers, ENYF staff, and community members about how to live well in this shared place.

### Authentic care

The literature on authentic care (Valenzuela, 1999), womanist caring (Beauboeuf-Lafontant, 2002), and other “politicized” understandings of care (Bartolomé, 2008) provides a context for understanding a major finding that emerged from this study, i.e., the presence of a kind of familial intimacy involving the affectionate scrutiny of habits, characteristics, and mannerism, or as one youth expressed, “people notic[ing] things about you at this job.” Although McKenzie and Blenkinsop (2006) have addressed issues of care in an outdoor adventure program, we are not aware of other empirical studies addressing this program element in urban environmental education. Authentic care offers a critical sociocultural perspective for understanding the intimacy developed among ENYF interns and staff, and how returning interns are initiated into and then help create this culture of care and caring at ENYF and in the community by leading first-year interns at the farm and market, and by their desire to effect change in the neighborhood related to food choices and access.

The ENYF culture of authentic care reflects Gruenewald's (2003) notions of how, when educators and learners recognize what needs to be transformed and what needs to be conserved, youth can re-story themselves and in so doing reinvent local social-ecological places. ENYF staff's caring also reflects political awareness as young people are cared for inside of individual relationships with attentiveness to the political landscape in which the child is living and to opportunities to enact social change (Beauboeuf-Lafontant, 2002). Intern stories reflect trends in Brooklyn and other urban centers with high rates of disconnected youth (MOA, 2012), where ENYF provides one counterpoint to non-caring spaces that youth often encounter. For participants, this opens the possibility of moving beyond “inactive caring” (McKenzie, 2006), in which students care about the environment (or their community) but feel unable to make positive change. The care modeled by staff is later assumed by returning interns who practice leadership at ENYF by caring for first-year interns, creating a feedback between the theme of practicing leadership and the belonging and being pushed themes that comprise authentic care.

### Critical consciousness

Drawing from Gruenewald's (2003) critical pedagogy of place, Smith (2007) describes how reinhabitation, or collective restoration and related stewardship activities, is more readily achieved in place-based education than decolonization, which involves developing a critical consciousness and addressing systemic injustice. At ENYF, reinhabitation entailed complex tasks, such as organizing and tracking multiple growers' produce at a farmers' market. Decolonization entailed struggling with larger issues related to organic wastes, food systems, and nutrition and food access in their community. The interns' narratives demonstrate they were able to accomplish the complex tasks, and that they were beginning to develop a critical consciousness as they struggled with more nuanced scientific and policy concepts. That the interns were beginning to think critically about larger issues may be due to the program's focus on food justice, which provides opportunities for interns to participate in and later lead workshops related to food deserts and similar issues. ENYF interns also participate in community organizing efforts and attend youth-centered conferences. In short, ENYF intern narratives describe how an urban environmental education program that challenges youth within a safe setting can provide affordances for youth to develop competence while demonstrating critical thinking and consciousness. This critical consciousness allows learners to perceive, understand, and potentially counter oppressive systems and structures (Freire, 1973).

### Affordances

In addition to using socio-environmental affordances such as adult mentors and urban gardening to develop their own assets, returning interns shape their larger program and community through mentoring new interns, educating adults, and providing access to fresh food for neighbors. In so doing, they extend notions of “shaping affordances” beyond manipulating nature (e.g., turning a stick into a play object, Said, 2012), and designing or planning infrastructure (Kyttä, 2002; Kyttä et al., 2004; Rudd et al., 2017), to encompass creating a public good by changing the physical and social landscape of their neighborhood. Playing an active role in civil society is in itself an affordance that leads to positive youth development outcomes, including a new sense of self and contribution to one's community (Lewis-Charp et al., 2003; Lerner et al., 2005), while engaging youth in addressing structural issues integral to social justice youth development (Ginwright and James, 2002; Sukarieh and Tannock, 2011).

## Conclusion

Although interns reported positive relationships with adults and peers outside of their ENYF activities, the program stood out as providing socio-environmental affordances that integrate access to natural areas like gardens and opportunities for revitalizing communities and reinventing one's story. Thus, ENYF provides affordances for positive youth development through a transactional approach, where an affordance is understood as a non-deterministic *in-situ* precondition for human activity, enabled by interactions of individual abilities with material and social features of the surrounding environment (Barthel and Kyttä, 2016). Youth at ENYF successfully address the challenges of growing produce and managing a farmers' market and demonstrate critical thinking and an emerging critical consciousness through posing questions about food security in an under-resourced, urban community of color. ENYF returning interns in turn become part of the social affordances for the development of assets among new interns, and through their composting, food production, and farmers' market activities, create affordances for the larger community. We attribute these results to the ENYF program design and leaders who create long-term repeat experiences, safe spaces, and appropriate challenges for youth. Although these outcomes are not universal across all urban environmental action intern programs, they do suggest that through attention to positive youth development, affordances, authentic care, and critical pedagogy of place, program leaders can foster youth assets that also contribute to socio-environmental affordances for additional youth and the broader community.

## Ethics statement

This study was approved by the Institutional Review Board of Cornell University and by East New York Farms. Informed consent using a Cornell IRB written form was obtained from all individual participants included in the study and from their parents for participants younger than 18 years.

## Author contributions

JD conducted the field work, analyzed data, and wrote a Master's thesis based on this work. MK was JD's Master's committee chair and took the lead in revising the thesis for publication.

### Conflict of interest statement

The authors declare that the research was conducted in the absence of any commercial or financial relationships that could be construed as a potential conflict of interest.

## Footnotes

^1^ENYF (2017). *East New York Farms!* Available online at: https://ucceny.org/enyf/.
